# Ambient particulate-phase polycyclic aromatic hydrocarbon mixtures and gastrointestinal disease prevalence in China: a population-based cross-sectional study

**DOI:** 10.1265/ehpm.26-00006

**Published:** 2026-03-31

**Authors:** Linwei Yao, Xi Wang, Yong Shen, Haobo Zhong

**Affiliations:** 1 Guangdong Medical University, Zhanjiang, Guangdong 524023, China; 2 The First People’s Hospital of Huizhou, Huizhou, Guangdong 516003, China

**Keywords:** Polycyclic aromatic hydrocarbons (PAHs), Mixture exposure, Gastrointestinal disease, CHARLS, Effect modification

## Abstract

**Background:**

Evidence regarding the association between ambient polycyclic aromatic hydrocarbon (PAH) mixtures and non-neoplastic gastrointestinal diseases in general populations remains limited. This study examined whether provincial ambient particulate-phase PAH burden was associated with prevalent gastrointestinal disease among middle-aged and older adults in China.

**Methods:**

Provincial annual mean concentrations of particulate-phase PAHs for 2015 were compiled from published monitoring studies across 12 Chinese provinces and linked to participants in the 2015 wave of the China Health and Retirement Longitudinal Study. Gastrointestinal disease was defined as a self-reported physician diagnosis and/or current treatment, excluding tumors and cancer. Using multivariable logistic regression, we estimated odds ratios and 95% confidence intervals for prevalent gastrointestinal disease per doubling (log2) of total PAHs and of 17 individual PAHs, adjusting for demographic, socioeconomic, and health-related covariates. The Benjamini–Hochberg procedure controlled the false discovery rate. Likelihood ratio tests for interaction were used to assess effect modification.

**Results:**

Of 3,671 adults aged 45 years or older, 1,056 (28.8%) reported a gastrointestinal disease. In fully adjusted models, each doubling of total PAH concentration was associated with increased odds of gastrointestinal disease (odds ratio 1.07, 95% confidence interval 1.02–1.12; false discovery rate–adjusted q = 0.014). Following multiple-comparison correction, naphthalene, fluorene, phenanthrene, and pyrene each demonstrated consistent positive associations. The association for total PAHs was more pronounced among non-drinkers (odds ratio 1.43, 95% confidence interval 1.19–1.71; P for interaction = 0.015) and rural residents (odds ratio 1.37, 95% confidence interval 1.15–1.65; P for interaction = 0.035).

**Conclusions:**

Higher provincial ambient particulate-phase PAH burden was associated with a greater prevalence of self-reported non-neoplastic gastrointestinal disease among middle-aged and older Chinese adults, with potential heterogeneity by residence and alcohol consumption. Given the cross-sectional design and province-level exposure assignment, longitudinal studies with individual-level exposure assessment and validated outcomes are needed.

**Supplementary information:**

The online version contains supplementary material available at https://doi.org/10.1265/ehpm.26-00006.

## Graphical Abstract

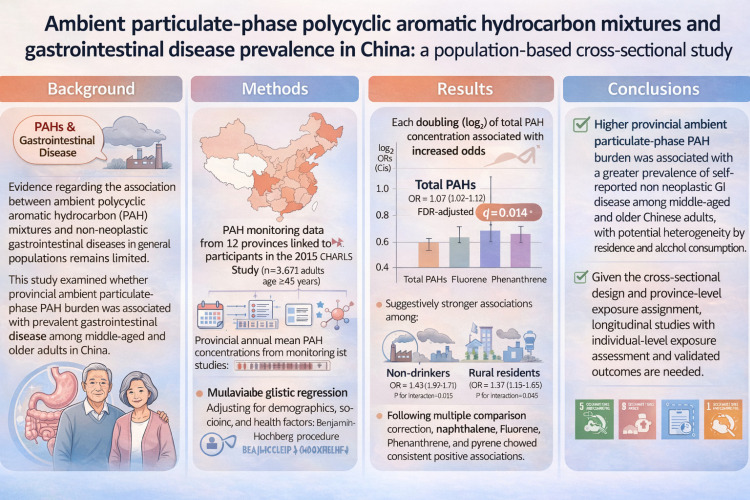

## 1. Introduction

Gastrointestinal conditions are a common and persistent health burden in middle-aged and older adults, encompassing a range of non-neoplastic disorders such as gastroesophageal reflux disease, peptic ulcer disease, inflammatory bowel disease, and functional gastrointestinal disorders [1, 2]. These conditions diminish quality of life, elevate healthcare utilization and medication needs, and often co-occur with malnutrition, sleep disturbances, and multisystem comorbidities, collectively contributing to health decline [3, 4]. Age-related declines in mucosal repair capacity, immune homeostasis, and neuroendocrine regulation of the intestinal barrier increase susceptibility to gastrointestinal disorders [5]. Concurrent multimorbidity and polypharmacy can further heighten vulnerability to external environmental stressors [6, 7]. Consequently, identifying modifiable environmental risk factors is essential for mitigating the gastrointestinal disease burden and promoting healthy aging.

Polycyclic aromatic hydrocarbons (PAHs) are ubiquitous persistent organic pollutants formed primarily through the incomplete combustion of carbonaceous materials [8, 9]. Their major sources encompass traffic and industrial emissions, residential and commercial cooking and heating, solid-fuel combustion, tobacco smoke, and high-temperature food preparation [10, 11]. Middle-aged and older adults often experience sustained exposure in both outdoor and indoor microenvironments, including via household cooking and heating activities [12], with long-term, low-dose exposure typically arising from multiple sources [13]. In contrast to conventional metrics like particulate matter mass, PAHs constitute a toxicologically relevant component of combustion-derived particles. Nevertheless, population-based evidence connecting ambient PAH exposure, especially to complex mixtures—to non-neoplastic gastrointestinal outcomes is still limited.

Mechanistically, PAHs are metabolically activated to reactive intermediates that promote oxidative stress and inflammatory responses, which compromise epithelial integrity and mucosal barrier function [14–16]. These pollutant-induced alterations in the mucosal immune microenvironment can increase intestinal permeability, impair tissue repair, and disrupt the composition and metabolic function of the gut microbiota, ultimately affecting intestinal peristalsis and gut–brain axis signaling [17, 18]. While epidemiologic studies have predominantly focused on cancer and cardiopulmonary outcomes, evidence linking PAHs to non-neoplastic gastrointestinal diseases (GIDs) remains limited and inconsistent [19–21]. Prior studies have also been constrained by non-representative samples, heterogeneous exposure assessment, and inadequate control for key confounders such as socioeconomic status and lifestyle [22, 23]. Given the correlated nature of PAH exposures, evaluating multiple compounds simultaneously may better capture the overall exposure burden.

Using data from the 2015 wave of the China Health and Retirement Longitudinal Study (CHARLS), a nationally representative survey of Chinese adults aged ≥45 years, we linked province-level annual mean particulate-phase PAH concentrations to individual health and demographic records [24]. We examined associations of total PAHs and 17 individual PAH compounds with the prevalence of self-reported, physician-diagnosed and/or treated non-neoplastic gastrointestinal disease and assessed potential effect modification by key sociodemographic and behavioral factors. This study aims to generate hypotheses and inform prevention strategies regarding combustion-related air pollution and gastrointestinal health in middle-aged and older adults.

## 2. Methods

### 2.1 Polycyclic aromatic hydrocarbon exposure data

We identified ambient particulate-phase PAH concentrations measured in Chinese cities in 2015 through searches of Web of Science, ScienceDirect, SpringerLink, and the China National Knowledge Infrastructure (CNKI). The search strategy combined terms for PAHs, particulate matter, atmospheric monitoring, and China [25]. For the 17 target PAH compounds, we extracted reported concentrations and aggregated available city-level measurements to calculate provincial annual mean concentrations for the 12 provinces with data. Additional details on data sources, extraction methods, and geographic coverage are provided in Supplementary Table S1 and Fig. S1.

### 2.2 Study population

**Fig. 1:**
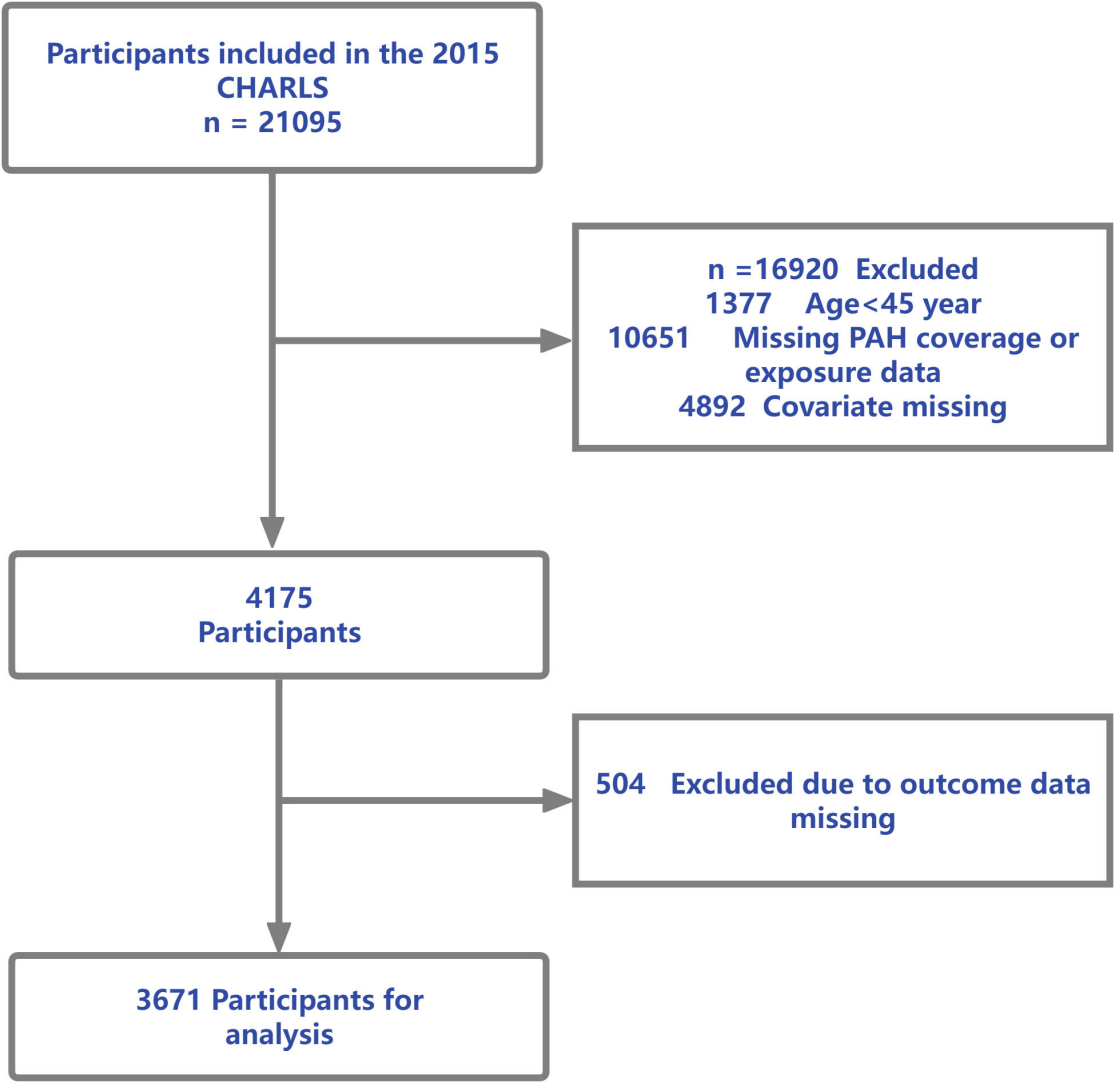
Flow diagram of participant selection from the 2015 CHARLS

We utilized cross-sectional data from the 2015 wave of CHARLS, a nationally representative survey of Chinese residents aged 45 years and older [24]. Provincial annual mean concentrations of particulate-phase PAHs for 2015 were linked to individual CHARLS records based on each participant’s province of residence. Eligible participants were at least 45 years old and had non-missing province information, complete data on gastrointestinal disease outcomes, and complete covariate data. We excluded individuals with missing outcome data, missing covariates, or residences in provinces lacking PAH exposure estimates. The final analytical sample comprised 3,671 participants (Fig. 1).

### 2.3 Assessment of gastrointestinal diseases

Gastrointestinal disease was defined based on self-reported physician diagnosis and current treatment status, in line with earlier research. Participants reported whether a physician had ever diagnosed them with a gastrointestinal disease, excluding tumors or cancer. Current treatment status was ascertained by asking if they were receiving treatment for a gastrointestinal disease or its complications [26]. The survey did not distinguish between specific digestive disorders, such as ulcerative colitis or peptic ulcer disease.

### 2.4 Covariates

To minimize potential confounding, we included several covariates in the analysis. We considered participants’ age, sex, and place of residence. Educational attainment was categorized as primary school or less, including no formal schooling, versus secondary school or higher. Marital status was classified as married and cohabiting, married but living apart, or other. Smoking status was dichotomized as ever versus never. Alcohol consumption was categorized by past-year drinking frequency as none, less than once per month, or at least once per month. Household cooking fuel was grouped as clean fuel or solid fuels. Waist circumference was also included as a covariate [24].

### 2.5 Statistical analysis

Logistic regression was used to assess associations between prevalent gastrointestinal disease and each of the 17 individual PAH compounds, as well as total PAHs. PAH concentrations were log2-transformed to interpret effect estimates as corresponding to a doubling in exposure. Three models were fitted: an unadjusted Model 1; Model 2, adjusted for age, sex, residence, education, and marital status; and Model 3, which additionally adjusted for smoking status, alcohol consumption, waist circumference, and household cooking fuel type. Odds ratios (ORs) and their 95% confidence intervals (CIs) are presented. Statistical significance was defined as a two-sided P value < 0.05. To account for multiple comparisons across PAH compounds in Model 3, we applied the Benjamini–Hochberg procedure to control the false discovery rate (FDR) and report q values.

### 2.6 Subgroup analysis

We examined potential effect modification by sex, residence, educational attainment, marital status, smoking status, and alcohol consumption. Stratified analyses were performed by fitting the fully adjusted model (Model 3) within each subgroup. To formally test interaction, we introduced exposure-by-subgroup product terms into Model 3 and used likelihood ratio tests to compare models with and without these terms. The corresponding P values for interaction are presented in Supplementary Table S2.

## 3. Results

### 3.1 Demographics of participants and initial profile

The analysis comprised 3,671 participants, including 1,056 with and 2,615 without a reported gastrointestinal disease. As presented in Table 1, the overall mean age was 62.1 years (SD 9.8), with no significant difference between the two groups (P = 0.488). A higher proportion of women was observed in the gastrointestinal disease group (55.4% vs. 48.0%, P < 0.001). Most participants lived in rural areas (61.4%), a distribution that was slightly more pronounced among those with gastrointestinal disease (64.2% vs. 60.2%, P = 0.028). Educational attainment also differed, as participants with gastrointestinal disease were more likely to have a primary school education or less (68.8% vs. 63.5%, P = 0.003), whereas marital status was comparable between groups (P = 0.349). Those with gastrointestinal disease were more frequently non-smokers (56.3% vs. 52.4%, P = 0.033) and non-drinkers (68.5% vs. 62.2%, P = 0.002), but the type of cooking fuel used did not differ significantly (P = 0.064). Waist circumference was lower in the gastrointestinal disease group (86.9 ± 10.5 cm vs. 88.5 ± 11.1 cm, P < 0.001). Among the PAH indicators, the distributions of Nap, Flu, Ace, Phe, Ant, Fla, Pyr, and BkF differed between groups (all P < 0.05), while the remaining PAH compounds and total PAH levels showed no significant difference (all P > 0.05).

**Table 1:** Baseline demographic characteristics, PAH exposure, and lifestyle indicators were stratified according to gastrointestinal disease status

	**level**	**Gastrointestinal diseases**				**SMD**

		**Overall**	**No**	**Yes**	**P**	
N		3671	2615	1056		
Age (mean (SD))		62.1 (9.8)	62.1 (10.0)	62.3 (9.5)	0.488	0.026
Sex (%)	Male	1831 (49.9)	1360 (52.0)	471 (44.6)	<0.001	0.149
	Female	1840 (50.1)	1255 (48.0)	585 (55.4)		
Residence (%)	Urban	1418 (38.6)	1040 (39.8)	378 (35.8)	0.028	0.082
	Rural	2253 (61.4)	1575 (60.2)	678 (64.2)		
Education Status (%)	Elementary school or below	2387 (65.0)	1661 (63.5)	726 (68.8)	0.003	0.111
	Middle school or above	1284 (35.0)	954 (36.5)	330 (31.2)		
Marital status (%)	Married and living with a spouse	2658 (72.4)	1910 (73.0)	748 (70.8)	0.349	0.053
	Married but living without spouse	204 (5.6)	145 (5.5)	59 (5.6)		
	other	809 (22.0)	560 (21.4)	249 (23.6)		
Smoking Status (%)	Non-smoker	1965 (53.5)	1370 (52.4)	595 (56.3)	0.033	0.079
	Smoker	1706 (46.5)	1245 (47.6)	461 (43.7)		
Drinking_Status (%)	Drink but less than once a month	342 (9.3)	256 (9.8)	86 (8.1)	0.002	0.132
	Drink more than once a month	979 (26.7)	732 (28.0)	247 (23.4)		
	Non-drinker	2350 (64.0)	1627 (62.2)	723 (68.5)		
Cooking Fuel (%)	Clean fuel	2250 (61.3)	1628 (62.3)	622 (58.9)	0.064	0.069
	Solid fuel	1421 (38.7)	987 (37.7)	434 (41.1)		
Waist circumference (mean (SD))		88.0 (10.9)	88.5 (11.1)	86.9 (10.5)	<0.001	0.148
Nap (median [IQR])		1.1 [0.9, 1.5]	1.0 [0.9, 1.5]	1.1 [0.9, 1.7]	0.001	0.101
Flu (median [IQR])		1.1 [0.2, 2.2]	0.3 [0.2, 2.2]	1.1 [0.2, 2.3]	0.001	0.116
Ace (median [IQR])		0.5 [0.2, 1.6]	0.5 [0.2, 1.6]	0.5 [0.2, 1.6]	0.012	0.077
Phe (median [IQR])		1.7 [0.6, 6.9]	1.5 [0.6, 6.9]	1.7 [1.0, 6.9]	0.005	0.086
Ant (median [IQR])		0.3 [0.2, 2.1]	0.3 [0.2, 2.1]	0.5 [0.2, 2.1]	0.034	0.077
Acy (median [IQR])		1.1 [0.4, 1.3]	1.1 [0.4, 1.3]	1.1 [0.4, 1.3]	0.753	0.036
Fla (median [IQR])		3.1 [0.6, 7.1]	3.1 [0.6, 7.1]	3.1 [1.5, 7.1]	0.037	0.054
Pyr (median [IQR])		3.7 [0.5, 5.2]	3.7 [0.5, 5.2]	3.7 [3.4, 5.2]	0.03	0.063
Chr (median [IQR])		4.7 [0.7, 6.2]	4.7 [0.7, 6.2]	4.7 [1.4, 6.2]	0.307	0.042
BaA (median [IQR])		3.6 [0.6, 5.1]	3.6 [0.6, 5.1]	3.6 [1.2, 5.1]	0.069	0.055
BbF (median [IQR])		5.1 [1.0, 11.6]	5.1 [1.0, 11.6]	5.1 [1.0, 11.6]	0.794	0.011
BkF (median [IQR])		2.5 [0.8, 9.0]	2.5 [0.8, 9.0]	2.5 [1.2, 9.0]	0.011	0.076
BaP (median [IQR])		3.1 [0.9, 4.1]	3.1 [0.9, 4.1]	3.1 [0.9, 4.1]	0.211	0.035
DahA (median [IQR])		1.1 [0.2, 1.3]	1.1 [0.2, 1.3]	1.1 [0.2, 1.3]	0.459	0.018
BghiP (median [IQR])		5.5 [0.9, 7.3]	5.5 [0.9, 7.3]	5.5 [0.9, 7.3]	0.551	0.023
IcdP (median [IQR])		6.1 [0.8, 8.2]	6.1 [0.8, 8.2]	6.1 [0.8, 8.2]	0.177	0.036
Total_PAHs (median [IQR])		52.9 [8.6, 75.9]	52.9 [8.6, 75.9]	52.9 [31.9, 75.9]	0.113	0.056

### 3.2 PAH concentration and comparative group analysis

As summarized in Table 1, the concentrations of several individual PAHs—including Nap, Flu, Ace, Phe, Ant, Fla, Pyr, and BkF—differed significantly between participants with and without gastrointestinal disease (all P < 0.05). In contrast, no significant differences were observed for Acy, Chr, BaA, BbF, BaP, DahA, BghiP, IcdP, or total PAHs (all P > 0.05). These findings indicate that the exposure profiles of specific PAH constituents vary according to gastrointestinal disease status.

### 3.3 PAHs exposure correlation with gastrointestinal disease risk

**Fig. 2:**
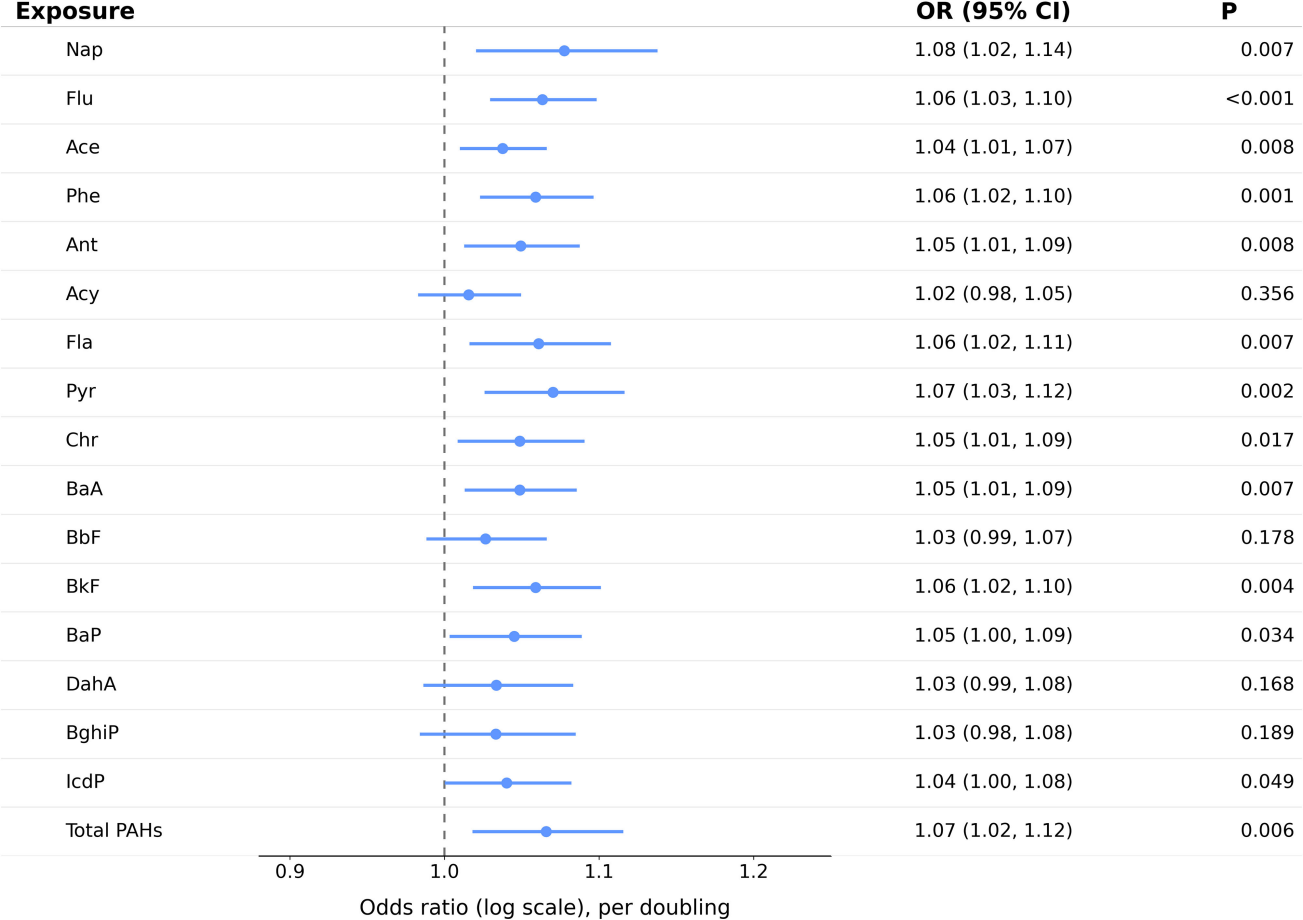
Associations between ambient particulate PAH indicators and prevalent gastrointestinal diseases Odds ratios (95% CIs) from multivariable logistic regression Models 1–3; Model 3 q-values were FDR-adjusted

We assessed the associations between PAH exposure and gastrointestinal disease using multivariable logistic regression in three sequential models. Model 1 was unadjusted, Model 2 adjusted for demographic and socioeconomic covariates, and Model 3 further adjusted for smoking, alcohol consumption, waist circumference, and household cooking fuel. As illustrated in Fig. 2 and Table 2, several PAHs exhibited persistent positive associations after adjustment. Nap was associated with higher disease odds in Models 1 and 2 and remained significant in Model 3 (OR = 1.08, 95% CI: 1.02–1.14; P = 0.007). Flu and Phe also showed consistent positive associations across all models. Pyr, Ace, Ant, BaA, BkF, and Fla were significantly associated with disease in each model, with Model 3 ORs ranging from 1.04 to 1.07 (all P < 0.008; q = 0.014). Chr and BaP were not significant in Models 1 or 2 but became significant in Model 3 (Chr: OR = 1.05, 95% CI: 1.01–1.09, P = 0.017, q = 0.027; BaP: OR = 1.05, 95% CI: 1.00–1.09, P = 0.034, q = 0.049). IcdP was borderline significant in Model 3 (OR = 1.04, 95% CI: 1.00–1.08, P = 0.049) but did not retain significance after FDR correction (q = 0.065). Total PAHs were significantly associated with disease in all models, most strongly in Model 3 (OR = 1.07, 95% CI: 1.02–1.12; P = 0.006; q = 0.014). In summary, several PAHs, including Nap, Flu, Pyr, and total PAHs—were consistently associated with elevated odds of gastrointestinal disease following multivariable adjustment.

**Table 2:** Multivariable logistic regression results for prevalent gastrointestinal diseases associated with ambient particulate PAH indicators

**Exposure**	**Model 1** **(OR, 95%CI)**	**Model 2** **(OR, 95%CI)**	**Model 3** **(OR, 95%CI)**	**Model 3 q** **(FDR)**
Flu	1.05 (1.02, 1.09) P = 0.001	1.05 (1.02, 1.09) p = 0.001	1.06 (1.03, 1.10) P < 0.001	0.003
Phe	1.05 (1.01, 1.08) P = 0.01	1.05 (1.01, 1.08) P = 0.008	1.06 (1.02, 1.10) P = 0.001	0.009
Pyr	1.05 (1.01, 1.10) P = 0.013	1.05 (1.01, 1.10) P = 0.013	1.07 (1.03, 1.12) P = 0.002	0.009
Ace	1.03 (1.00, 1.06) P = 0.026	1.03 (1.00, 1.06) P = 0.031	1.04 (1.01, 1.07) P = 0.008	0.014
Ant	1.04 (1.00, 1.08) P = 0.033	1.04 (1.00, 1.08) P = 0.027	1.05 (1.01, 1.09) P = 0.008	0.014
BaA	1.04 (1.00, 1.07) P = 0.035	1.04 (1.00, 1.07) P = 0.042	1.05 (1.01, 1.09) P = 0.007	0.014
BkF	1.05 (1.01, 1.09) P = 0.016	1.05 (1.01, 1.09) P = 0.019	1.06 (1.02, 1.10) P = 0.004	0.014
Fla	1.04 (1.00, 1.09) P = 0.045	1.05 (1.00, 1.09) P = 0.041	1.06 (1.02, 1.11) P = 0.007	0.014
Nap	1.06 (1.01, 1.12) P = 0.023	1.06 (1.00, 1.12) P = 0.037	1.08 (1.02, 1.14) P = 0.007	0.014
Chr	1.04 (1.00, 1.08) P = 0.065	1.03 (1.00, 1.07) P = 0.084	1.05 (1.01, 1.09) P = 0.017	0.027
BaP	1.03 (0.99, 1.07) P = 0.126	1.03 (0.99, 1.07) P = 0.133	1.05 (1.00, 1.09) P = 0.034	0.049
IcdP	1.03 (0.99, 1.07) P = 0.122	1.03 (0.99, 1.07) P = 0.148	1.04 (1.00, 1.08) P = 0.049	0.065
DahA	1.03 (0.98, 1.07) P = 0.267	1.02 (0.98, 1.07) P = 0.3	1.03 (0.99, 1.08) P = 0.168	0.201
BghiP	1.02 (0.98, 1.07) P = 0.327	1.02 (0.97, 1.07) P = 0.404	1.03 (0.98, 1.08) P = 0.189	0.201
BbF	1.02 (0.98, 1.06) P = 0.319	1.02 (0.98, 1.05) P = 0.393	1.03 (0.99, 1.07) P = 0.178	0.201
Acy	1.01 (0.98, 1.04) P = 0.529	1.01 (0.98, 1.04) P = 0.623	1.02 (0.98, 1.05) P = 0.356	0.356
Total PAHs	1.05 (1.00, 1.10) P = 0.034	1.05 (1.00, 1.10) P = 0.038	1.07 (1.02, 1.12) P = 0.006	0.014

### 3.4 Stratified subgroup analyses of PAH-related gastrointestinal condition heterogeneity

**Fig. 3:**
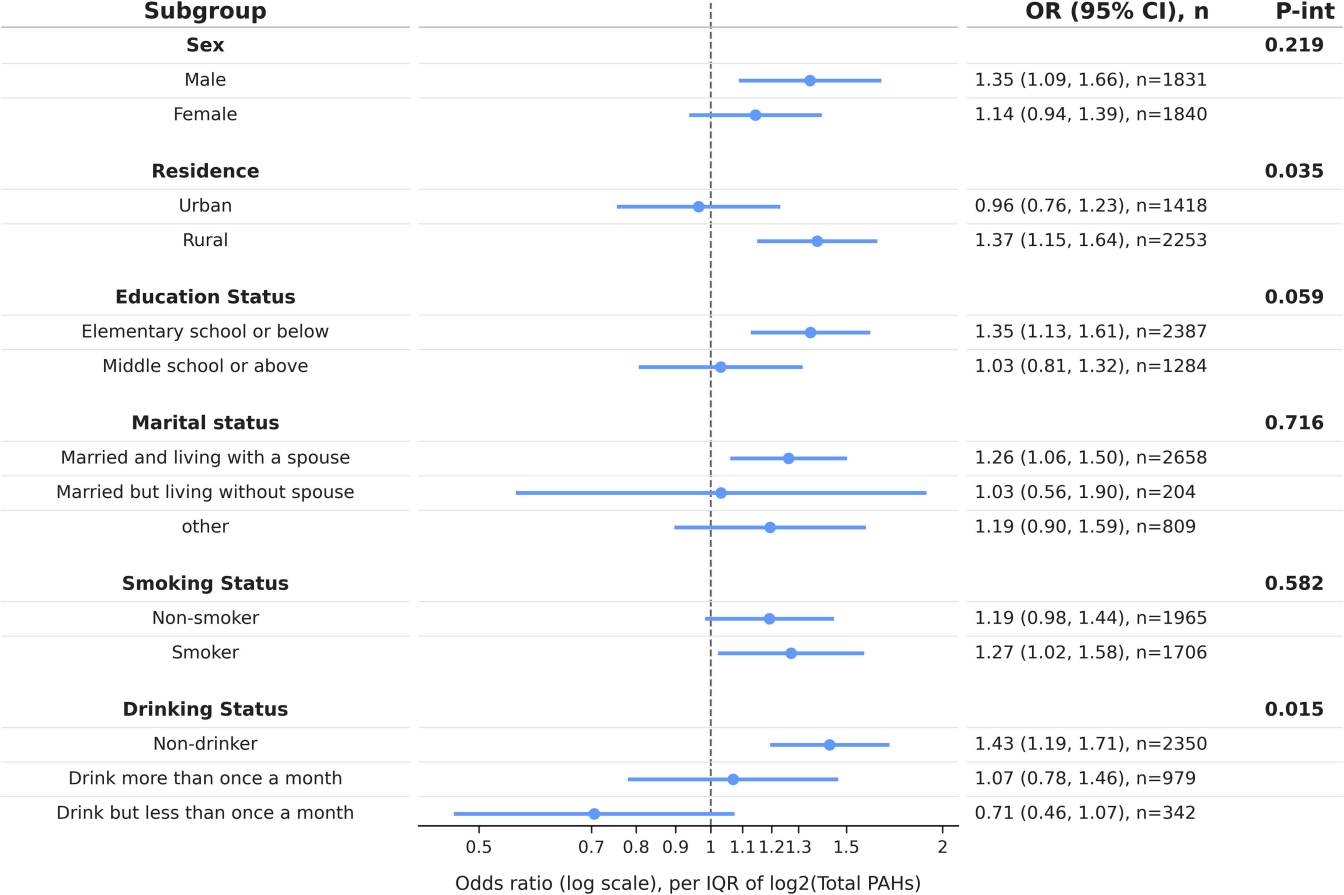
Subgroup analyses of PAH–gastrointestinal disease associations by demographic and behavioral factors Adjusted specific odds ratios (95% confidence intervals) based on Model 3

Stratified analyses were conducted by sex, residence, educational attainment, marital status, smoking status, and alcohol consumption to assess whether the associations differed across these subgroups (see Fig. 3 and Supplementary Fig. S2–S5). For each stratum, odds ratios (ORs) and 95% confidence intervals (CIs) per doubling in PAH concentrations were estimated to use the same modeling strategy as in the primary analysis. Interaction was evaluated by incorporating exposure-by-subgroup terms into Model 3 and applying likelihood ratio tests; the corresponding P values are presented in Supplementary Table S2.

Overall, the subgroup patterns indicated potential heterogeneity, although most interaction tests were not statistically significant, so these results should be interpreted as exploratory. Sex-stratified analyses suggested stronger associations in men for selected PAHs, with nominal evidence of interaction for Ace (P interaction = 0.021) and Ant (P interaction = 0.046). Urban–rural stratification indicated stronger associations among rural residents for overall PAH burden and several higher-molecular-weight PAHs, with a nominal interaction for total PAHs (P interaction = 0.035). Education-stratified analyses suggested stronger associations among participants with a primary school education or less for Pyr (P interaction = 0.040), whereas interaction testing provided limited evidence of robust modification by marital status or smoking status. Alcohol consumption showed the most consistent pattern of potential effect modification, with nominal interactions for total PAHs (P interaction = 0.015) and multiple individual PAHs. Given that subgroup analyses involve multiple comparisons, these findings should be regarded as hypothesis-generating and interpreted with caution; the P interaction values were not corrected for multiple testing (Supplementary Table S2).

## 4. Discussion

In this cross-sectional analysis of 3,671 middle-aged and older adults from CHARLS, linked to province-level ambient particulate-phase PAH concentrations compiled from 2015 monitoring studies, a higher PAH burden was associated with an increased prevalence of self-reported non-neoplastic gastrointestinal disease. The outcome definition, which encompassed physician-diagnosed gastrointestinal disease excluding tumors and current treatment status, captures clinically relevant morbidity in an aging population. In the fully adjusted model, each doubling of total PAHs corresponded to 7% higher odds of disease (OR = 1.07, 95% CI: 1.02–1.12; q = 0.014). Several individual PAHs, including naphthalene, fluorene, phenanthrene, and pyrene, also showed consistent positive associations after false discovery rate correction. These findings collectively support the hypothesis that combustion-related particulate PAH mixtures may contribute to gastrointestinal morbidity in middle-aged and older adults.

The modest effect size for total PAHs may still be important from a population-health perspective, given widespread exposure to combustion-related air pollution. Several analytical features support a mixture-centered interpretation. Sensitivity analyses indicated that the positive association persisted when exposure was expressed using alternative contrasts, such as IQR- and SD-based contrasts on the log2 scale, suggesting the inference was not driven by the choice of exposure unit. The convergence of signals across multiple-correlated PAHs further suggests the association is unlikely to be attributable to a single compound. Instead, total particulate-phase PAHs likely reflect an integrated burden from combustion-related mixtures, with individual PAHs serving as correlated indicators of shared sources and exposure pathways.

Our findings align with broader evidence that combustion-related exposures can affect gastrointestinal health. In a cohort of Chinese middle-aged and older adults, indoor solid-fuel use—a major source of PAHs and other combustion byproducts—was associated with a higher risk of non-neoplastic digestive diseases, supporting the plausibility of combustion mixtures as gastrointestinal stressors in comparable populations [12]. This study extends prior work by examining ambient particulate-phase PAHs as a chemically specific component of combustion-derived particles, offering greater toxicological specificity than studies relying on broader exposure proxies. Heterogeneity across studies is expected because gastrointestinal disease includes diverse phenotypes, and aggregated endpoints may dilute associations if only specific subtypes are pollution sensitive. Province-level annual mean exposure estimates do not capture within-province spatial variability, seasonal dynamics, or individual behaviors, likely introducing exposure measurement error that may bias estimates toward the null. Moreover, because PAHs covary with other combustion-related pollutants, these associations are best interpreted as reflecting a PAH-centered marker of combustion mixtures rather than an isolated causal effect of PAHs.

Several biological pathways plausibly link particulate-phase PAHs to gastrointestinal morbidity. PAHs originate from incomplete combustion and occur as complex environmental mixtures [27]. Inhaled particle-bound PAHs can be cleared via mucociliary transport and subsequently swallowed, and PAHs can also enter the gastrointestinal tract through contaminated food and water [28]. Following metabolic activation, PAHs generate reactive intermediates that induce oxidative stress, inflammatory signaling, and DNA damage [29]. These processes may exacerbate mucosal injury and inflammatory activity across a range of gastrointestinal conditions, consistent with the observed direction of association.

Intestinal barrier dysfunction provides a unifying mechanistic framework linking chemical mixtures to heterogeneous gastrointestinal outcomes. The mucosal barrier depends on tight junction integrity and immune homeostasis; its disruption increases permeability, facilitates microbial translocation, and sustains inflammation [30, 31]. PAH-induced oxidative stress may directly impair epithelial integrity and may also activate xenobiotic-sensing pathways. The aryl hydrocarbon receptor (AHR) integrates exogenous chemical cues with mucosal immunity and inflammatory programming [32]. Additional xenobiotic-responsive nuclear receptors, including PXR and CAR, shape microbial ecology and inflammatory tone, potentially influencing susceptibility to environmental mixtures [33]. Experimental evidence supports this framework: ingestion of urban particulate matter increases intestinal permeability, triggers local inflammatory responses, and alters gut microbial composition [34]. Air pollution has also been proposed to influence inflammatory disease through microbiome-mediated mechanisms [35], and PAH-specific exposure, such as oral benzo[a]pyrene, disrupts microbial communities and affects the intestinal epithelium in animal models [36]. Collectively, these findings are consistent with our epidemiologic observations and underscore the need for biomarker-based validation to strengthen causal inference.

Effect modification analyses suggested potential heterogeneity by residence and alcohol consumption, with stronger associations among rural residents and non-drinkers, although some subgroup estimates were imprecise. The rural pattern may reflect higher co-exposures, including household combustion sources, fewer opportunities for exposure reduction, and differences in healthcare access and comorbidity profiles. The alcohol-related heterogeneity should be interpreted cautiously and not as evidence of a causal protective effect of alcohol; it may instead reflect differences in lifestyle, diet, medication use, or diagnosis and reporting. Overall, these subgroup findings are hypothesis-generating and motivate additional research to identify vulnerable populations and modifiable exposure contexts.

Several limitations merit consideration. Gastrointestinal disease was identified using self-reported physician diagnosis and current treatment status, without adjudication of clinical subtypes; while this approach is common in large-scale surveys, it may introduce outcome misclassification and limits etiological specificity. Exposure assessment relied on province-level annual mean particulate-phase PAHs compiled from published studies, which cannot capture fine-scale spatial variability or seasonal patterns and may be affected by methodological heterogeneity across source studies, leading to measurement error and residual regional confounding. Because co-pollutants and other particle constituents correlated with PAHs were not jointly modeled, the estimates should be interpreted as reflecting a PAH-centered marker of combustion mixtures rather than an isolated effect of PAHs. In addition, exclusions due to limited exposure coverage and missing covariates may have introduced selection bias and reduced generalizability. Finally, the cross-sectional design precludes firm conclusions about temporality and raises the possibility of reverse causation.

Despite these limitations, this study provides a rationale for future research and prevention. Longitudinal designs with fine-scale, geocoded exposure surfaces are needed to better capture within-province variability and establish temporality, while evaluating PAH mixtures alongside correlated co-pollutants using multipollutant and mixture modeling approaches. Validation of gastrointestinal outcomes using clinical records or standardized symptom instruments would improve reliability and interpretability. Integrating biomarkers—such as urinary hydroxylated PAH metabolites, hemoglobin adducts reflecting longer-term exposure, markers of oxidative stress and inflammation, and measures of intestinal permeability and gut microbiome profiles—would enable direct tests of mechanistic hypotheses and strengthen causal inference [37]. From a public health perspective, reducing combustion-related emissions and mitigating exposure in vulnerable communities may yield co-benefits for gastrointestinal and cardiopulmonary health.

## 5. Conclusions

This cross-sectional analysis indicates that higher ambient particulate-phase PAH burden at the provincial level is associated with an increased prevalence of self-reported non-neoplastic gastrointestinal disease in middle-aged and older Chinese adults. Total PAHs and specific constituents (Nap, Flu, Phe, and Pyr) maintained positive associations following multivariable adjustment and FDR correction, with stronger associations observed among rural residents and non-drinkers. These findings justify further investigation into combustion-related PAH mixtures as potential contributors to gastrointestinal morbidity and highlight the necessity for refined exposure assessment and outcome validation in subsequent studies.

## Abbreviations

PAH: Polycyclic aromatic hydrocarbon
GIDs: Non-neoplastic gastrointestinal diseases
PAHs: Polycyclic aromatic hydrocarbons
CHARLS: China Health and Retirement Longitudinal Study
ORs: Odds ratios
CIs: Confidence intervals
log2: Log base 2
FDR: False discovery rate
OR: Odds ratio
CI: Confidence interval
q: q value (FDR-adjusted)
P-interaction: P value for interaction
CNKI: China National Knowledge Infrastructure
P: P value
SD: Standard deviation
IQR: Interquartile range
DNA: Deoxyribonucleic acid
AHR: Aryl hydrocarbon receptor
PXR: Pregnane X receptor
CAR: Constitutive androstane receptor
Nap: Naphthalene
Flu: Fluorene
Ace: Acenaphthene
Phe: Phenanthrene
Ant: Anthracene
Fla: Fluoranthene
Pyr: Pyrene
BkF: Benzo[k]fluoranthene
Acy: Acenaphthylene
Chr: Chrysene
BaA: Benzo[a]anthracene
BbF: Benzo[b]fluoranthene
BaP: Benzo[a]pyrene
DahA: Dibenzo[a,h]anthracene
BghiP: Benzo[g,h,i]perylene
IcdP: Indeno[1,2,3-cd]pyrene

## Supplementary information

**Additional file 1:** **Table S1** Participant selection and exclusion at each step. **Table S2** P for interaction in subgroup analyses (Model 3; per IQR increase in log2-transformed PAHs). **Table S3** Sensitivity analysis of Model 3 associations using province-clustered robust standard errors (CR2). **Supplementary Table S4.** Leave-one-province-out (LOPO) analysis for total PAHs (Model 3). **Figure S1** Provincial-level spatial distribution of annual mean PM2.5-bound particulate-phase PAH concentrations in China (2015). **Figure S2.** Subgroup analyses of the association between ambient particulate-phase total PAHs and prevalent non-neoplastic gastrointestinal diseases. **Figure S3.** Subgroup analyses of the association between ambient particulate-phase naphthalene (Nap) and prevalent non-neoplastic gastrointestinal diseases. **Figure S4.** Subgroup analyses of the association between ambient particulate-phase fluorene (Flu) and prevalent non-neoplastic gastrointestinal diseases. **Figure S5.** Subgroup analyses of the association between ambient particulate-phase acenaphthene (Ace) and prevalent non-neoplastic gastrointestinal diseases. **Figure S6.** Subgroup analyses of the association between ambient particulate-phase phenanthrene (Phe) and prevalent non-neoplastic gastrointestinal diseases. **Figure S7.** Subgroup analyses of the association between ambient particulate-phase anthracene (Ant) and prevalent non-neoplastic gastrointestinal diseases. **Figure S8.** Subgroup analyses of the association between ambient particulate-phase fluoranthene (Fla) and prevalent non-neoplastic gastrointestinal diseases. **Figure S9.** Subgroup analyses of the association between ambient particulate-phase pyrene (Pyr) and prevalent non-neoplastic gastrointestinal diseases. **Figure S10.** Subgroup analyses of the association between ambient particulate-phase benzo[k]fluoranthene (BkF) and prevalent non-neoplastic gastrointestinal diseases. **Figure S11.** Subgroup analyses of the association between ambient particulate-phase acenaphthylene (Acy) and prevalent non-neoplastic gastrointestinal diseases. **Figure S12.** Subgroup analyses of the association between ambient particulate-phase chrysene (Chr) and prevalent non-neoplastic gastrointestinal diseases. **Figure S13.** Subgroup analyses of the association between ambient particulate-phase benzo[a]anthracene (BaA) and prevalent non-neoplastic gastrointestinal diseases. **Figure S14.** Subgroup analyses of the association between ambient particulate-phase benzo[b]fluoranthene (BbF) and prevalent non-neoplastic gastrointestinal diseases. **Figure S15.** Subgroup analyses of the association between ambient particulate-phase benzo[a]pyrene (BaP) and prevalent non-neoplastic gastrointestinal diseases. **Figure S16.** Subgroup analyses of the association between ambient particulate-phase dibenzo[a,h]anthracene (DahA) and prevalent non-neoplastic gastrointestinal diseases. **Figure S17.** Subgroup analyses of the association between ambient particulate-phase benzo[g,h,i]perylene (BghiP) and prevalent non-neoplastic gastrointestinal diseases. **Figure S18.** Subgroup analyses of the association between ambient particulate-phase indeno[1,2,3-cd]pyrene (IcdP) and prevalent non-neoplastic gastrointestinal diseases.

## Declarations

### Data availability

The CHARLS datasets can be downloaded to the CHARLS homepage at http://charls.pku.edu.cn/en.
