# Supplementary material for: Ambient particulate-phase polycyclic aromatic hydrocarbon mixtures and gastrointestinal disease prevalence in China: a population-based cross-sectional study
Source: Environ Health Prev Med. 2026 Mar 31;31:25. doi: 10.1265/ehpm.26-00006 (PMC13057908; doi:10.1265/ehpm.26-00006)
Supplement: Supplementary file 1 — Additional file 1: Table S1 Participant selection and exclusion at each step. Table S2 P for interaction in subgroup analyses (Model 3; per IQR increase in log2-transformed PAHs). Table S3 Sensitivity analysis of Model 3 associations using province-clustered robust standard errors (CR2). Supplementary Table S4. Leave-one-province-out (LOPO) analysis for total PAHs (Model 3). Figure S1 Provincial-level spatial distribution of annual mean PM2.5-bound particulate-phase PAH concentrations in China (2015). Figure S2. Subgroup analyses of the association between ambient particulate-phase total PAHs and prevalent non-neoplastic gastrointestinal diseases. Figure S3. Subgroup analyses of the association between ambient particulate-phase naphthalene (Nap) and prevalent non-neoplastic gastrointestinal diseases. Figure S4. Subgroup analyses of the association between ambient particulate-phase fluorene (Flu) and prevalent non-neoplastic gastrointestinal diseases. Figure S5. Subgroup analyses of the association between ambient particulate-phase acenaphthene (Ace) and prevalent non-neoplastic gastrointestinal diseases. Figure S6. Subgroup analyses of the association between ambient particulate-phase phenanthrene (Phe) and prevalent non-neoplastic gastrointestinal diseases. Figure S7. Subgroup analyses of the association between ambient particulate-phase anthracene (Ant) and prevalent non-neoplastic gastrointestinal diseases. Figure S8. Subgroup analyses of the association between ambient particulate-phase fluoranthene (Fla) and prevalent non-neoplastic gastrointestinal diseases. Figure S9. Subgroup analyses of the association between ambient particulate-phase pyrene (Pyr) and prevalent non-neoplastic gastrointestinal diseases. Figure S10. Subgroup analyses of the association between ambient particulate-phase benzo[k]fluoranthene (BkF) and prevalent non-neoplastic gastrointestinal diseases. Figure S11. Subgroup analyses of the association between ambient particulate-phase acenaphth [file ehpm-31-025-s001.doc]

Supporting Information

Ambient particulate-phase polycyclic aromatic hydrocarbon mixtures and gastrointestinal disease prevalence in China: a population-based cross-sectional study

Linwei Yao^1^, Xi Wang^2,1, *^, Yong Shen^2^, Haobo Zhong^1,2^

^1^ Guangdong Medical University, Zhanjiang, Guangdong 524023, China.

^2^ Department of Gastrointestinal Surgery, Huizhou First Hospital, Huizhou, Guangdong 516003, China.

^*^ Corresponding author: Xi Wang, Department of Gastrointestinal Surgery, Huizhou First Hospital, Huizhou, Guangdong 516003, China.

E-mail: [hwangxi@126.com](mailto:hwangxi@126.com)

All authors have read and agreed to the published version of the manuscript.

**Table S1 Participant selection and exclusion at each step**

| **Selection step** | **N before** | **Excluded n (%)** | **N after** | **Rationale** |
| --- | --- | --- | --- | --- |
| Step1: Age >=45 | 21,095 | 1,377 (6.5%) | 19,718 | Excluded: age <45 years or missing age |
| Step2: Exposure available (province covered) | 19,718 | 10,651 (54.0%) | 9,067 | Excluded: province not covered by PAH data or missing exposure |
| Step3: Complete case for covariates | 9,067 | 4,892 (54.0%) | 4,175 | Excluded: missing covariates |
| Step4: Outcome defined (Yes/No only) | 4,175 | 504 (12.1%) | 3,671 | Excluded: outcome undefined |

**Table S2 P for interaction in subgroup analyses (Model 3; per IQR increase in log2-transformed PAHs)**

| Abbrev. | Exposure | N used | Sex | Residence | Education | Tobacco use | Alcohol consumption | Marital status |
| --- | --- | --- | --- | --- | --- | --- | --- | --- |
| Nap | Naphthalene | 3531 | 0.101 (df=1) | 0.425 (df=1) | 0.874 (df=1) | 0.627 (df=1) | 0.345 (df=2) | 0.525 (df=2) |
| Flu | Fluorene | 3531 | 0.108 (df=1) | 0.242 (df=1) | 0.915 (df=1) | 0.589 (df=1) | 0.055 (df=2) | 0.267 (df=2) |
| Ace | Acenaphthene | 3531 | 0.021 (df=1) | 0.353 (df=1) | 0.920 (df=1) | 0.262 (df=1) | 0.212 (df=2) | 0.590 (df=2) |
| Phe | Phenanthrene | 3671 | 0.090 (df=1) | 0.118 (df=1) | 0.052 (df=1) | 0.582 (df=1) | 0.094 (df=2) | 0.558 (df=2) |
| Ant | Anthracene | 3671 | 0.046 (df=1) | 0.112 (df=1) | 0.815 (df=1) | 0.208 (df=1) | 0.022 (df=2) | 0.524 (df=2) |
| Acy | Acenaphthylene | 3315 | 0.295 (df=1) | 0.480 (df=1) | 0.330 (df=1) | 0.901 (df=1) | 0.607 (df=2) | 0.611 (df=2) |
| Fla | Fluoranthene | 3671 | 0.142 (df=1) | 0.020 (df=1) | 0.051 (df=1) | 0.419 (df=1) | 0.011 (df=2) | 0.747 (df=2) |
| Pyr | Pyrene | 3671 | 0.292 (df=1) | 0.039 (df=1) | 0.040 (df=1) | 0.792 (df=1) | 0.013 (df=2) | 0.675 (df=2) |
| Chr | Chrysene | 3671 | 0.327 (df=1) | 0.037 (df=1) | 0.092 (df=1) | 0.648 (df=1) | 0.018 (df=2) | 0.678 (df=2) |
| BaA | Benzo[a]anthracene | 3671 | 0.196 (df=1) | 0.040 (df=1) | 0.141 (df=1) | 0.402 (df=1) | 0.010 (df=2) | 0.588 (df=2) |
| BbF | Benzo[b]fluoranthene | 3671 | 0.446 (df=1) | 0.071 (df=1) | 0.099 (df=1) | 0.773 (df=1) | 0.108 (df=2) | 0.468 (df=2) |
| BkF | Benzo[k]fluoranthene | 3671 | 0.086 (df=1) | 0.139 (df=1) | 0.301 (df=1) | 0.264 (df=1) | 0.035 (df=2) | 0.382 (df=2) |
| BaP | Benzo[a]pyrene | 3671 | 0.468 (df=1) | 0.024 (df=1) | 0.056 (df=1) | 0.602 (df=1) | 0.016 (df=2) | 0.400 (df=2) |
| DahA | Dibenzo[a,h]anthracene | 3671 | 0.143 (df=1) | 0.230 (df=1) | 0.822 (df=1) | 0.186 (df=1) | 0.129 (df=2) | 0.407 (df=2) |
| BghiP | Benzo[g,h,i]perylene | 3671 | 0.436 (df=1) | 0.022 (df=1) | 0.172 (df=1) | 0.540 (df=1) | 0.028 (df=2) | 0.356 (df=2) |
| IcdP | Indeno[1,2,3-cd]pyrene | 3671 | 0.384 (df=1) | 0.023 (df=1) | 0.127 (df=1) | 0.538 (df=1) | 0.018 (df=2) | 0.470 (df=2) |
| Total_PAHs | Total PAHs (sum of measured PAHs) | 3671 | 0.219 (df=1) | 0.035 (df=1) | 0.059 (df=1) | 0.582 (df=1) | 0.015 (df=2) | 0.716 (df=2) |

**Table S3 Sensitivity analysis of Model 3 associations using province-clustered robust standard errors (CR2)**

| **Exposure (log2-transformed)** | **n** | **Main OR (95% CI)** | **CR2 clustered OR (95% CI)** |
| --- | --- | --- | --- |
| Nap | 3531 | 1.08 (1.02–1.14) | 1.08 (0.97–1.19) |
| Flu | 3531 | 1.06 (1.03–1.10) | 1.06 (1.03–1.10) |
| Ace | 3531 | 1.04 (1.01–1.07) | 1.04 (0.99–1.08) |
| Phe | 3671 | 1.06 (1.02–1.10) | 1.06 (1.03–1.09) |
| Ant | 3671 | 1.05 (1.01–1.09) | 1.05 (1.01–1.09) |
| Acy | 3315 | 1.02 (0.98–1.05) | 1.02 (0.97–1.07) |
| Fla | 3671 | 1.06 (1.02–1.11) | 1.06 (1.02–1.11) |
| Pyr | 3671 | 1.07 (1.03–1.12) | 1.07 (1.04–1.10) |
| Chr | 3671 | 1.05 (1.01–1.09) | 1.05 (1.00–1.10) |
| BaA | 3671 | 1.05 (1.01–1.09) | 1.05 (1.02–1.08) |
| BbF | 3671 | 1.03 (0.99–1.07) | 1.03 (0.96–1.10) |
| BkF | 3671 | 1.06 (1.02–1.10) | 1.06 (1.02–1.10) |
| BaP | 3671 | 1.05 (1.00–1.09) | 1.05 (0.99–1.10) |
| DahA | 3671 | 1.03 (0.99–1.08) | 1.03 (0.97–1.10) |
| BghiP | 3671 | 1.03 (0.98–1.08) | 1.03 (0.95–1.13) |
| IcdP | 3671 | 1.04 (1.00–1.08) | 1.04 (0.98–1.10) |
| Total_PAHs | 3671 | 1.07 (1.02–1.12) | 1.07 (1.02–1.11) |

ORs correspond to a **doubling** in PAH concentrations (log2-transformed).

Model 3 adjusted for age, sex, residence, education, marital status, smoking, alcohol drinking, waist circumference, and cooking fuel.

CR2 indicates a small-sample corrected cluster-robust variance estimator with clustering at the **province** level (12 provinces).

| **Province left out** | **n** | **clusters** | **OR (95% CI)** |
| --- | --- | --- | --- |
| Anhui | 3260 | 11 | 1.07 (1.02–1.12) |
| Beijing | 3655 | 11 | 1.07 (1.02–1.12) |
| Gansu | 3455 | 11 | 1.05 (1.00–1.11) |
| Guangdong | 3292 | 11 | 1.07 (1.01–1.12) |
| Hebei | 3311 | 11 | 1.05 (1.00–1.11) |
| Henan | 3051 | 11 | 1.07 (1.02–1.13) |
| Heilongjiang | 3531 | 11 | 1.06 (1.02–1.11) |
| Jiangsu | 3290 | 11 | 1.07 (1.02–1.12) |
| Shandong | 2932 | 11 | 1.08 (1.03–1.13) |
| Shanghai | 3650 | 11 | 1.07 (1.02–1.12) |

**Supplementary Table S4. Leave-one-province-out (LOPO) analysis for total PAHs (Model 3)**

ORs correspond to a doubling in total PAHs (log2-transformed).

Model 3 covariate adjustment as in the main analysis.

Figure S1 Provincial-level spatial distribution of annual mean PM2.5-bound particulate-phase PAH concentrations in China (2015)


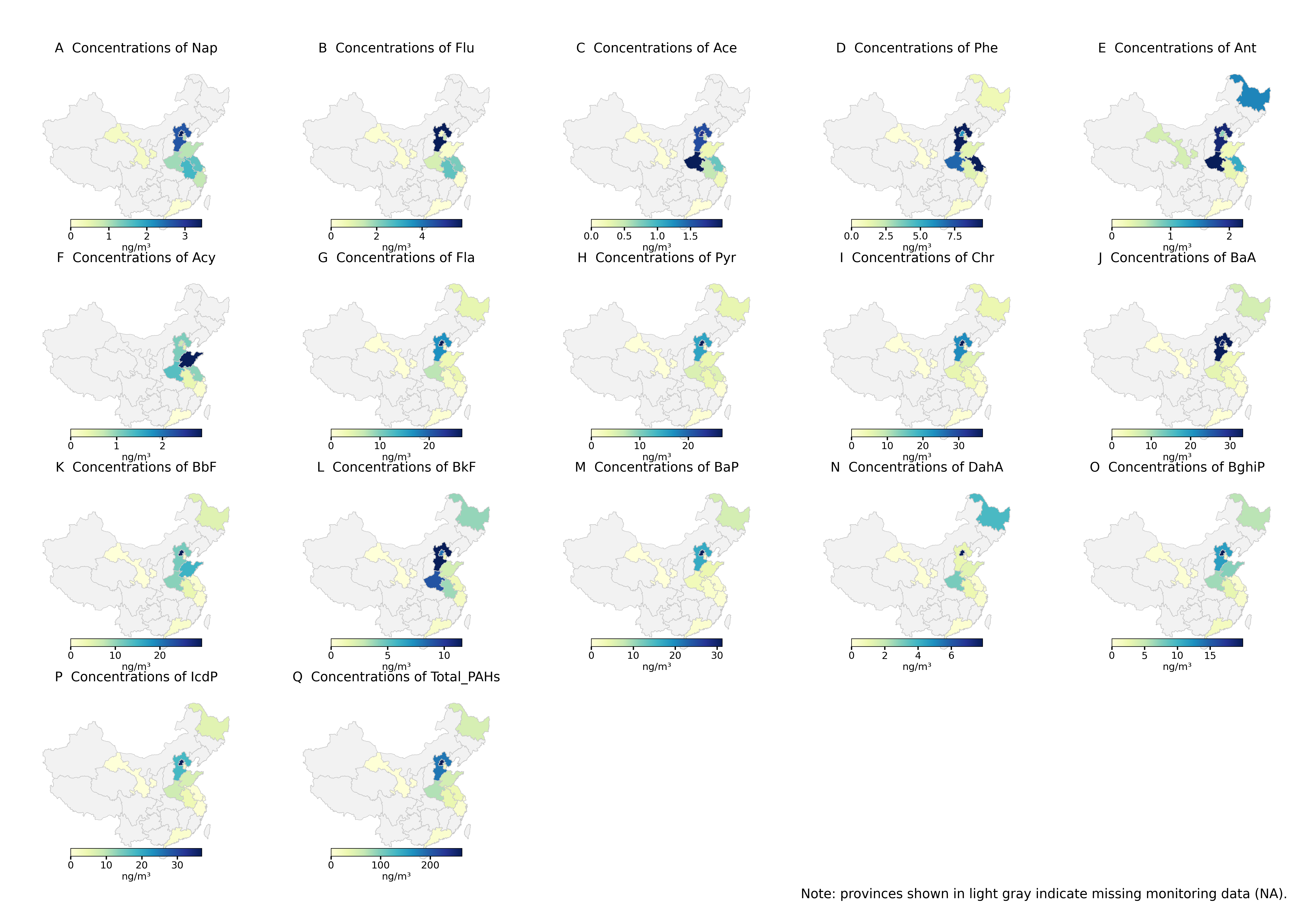
Panels (A–P) show individual PAHs and panel (Q) shows Total PAHs (ng/m³); provinces in light gray indicate missing monitoring data (NA).

**Supplementary Figures**


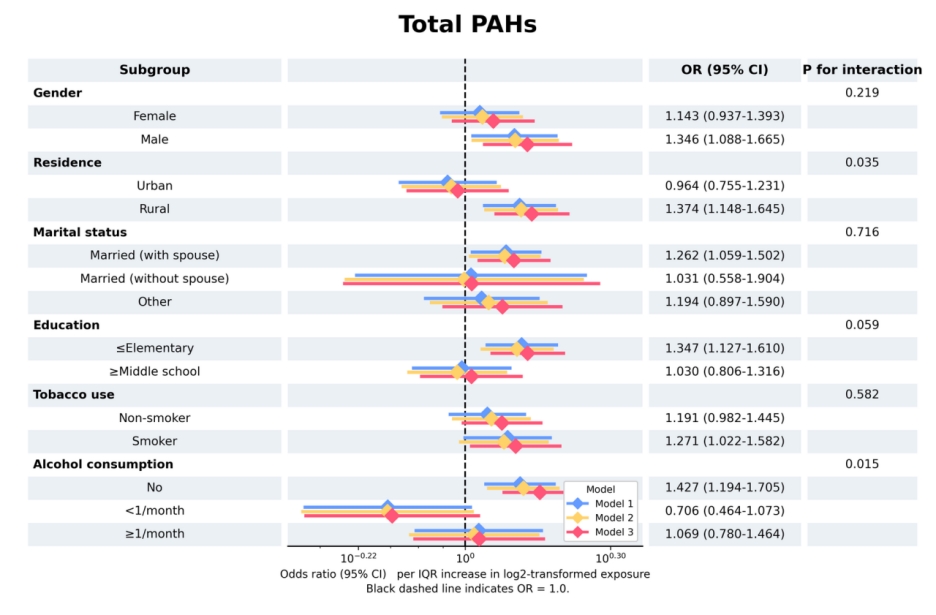


Figure S2. Subgroup analyses of the association between ambient particulate-phase total PAHs and prevalent non-neoplastic gastrointestinal diseases.


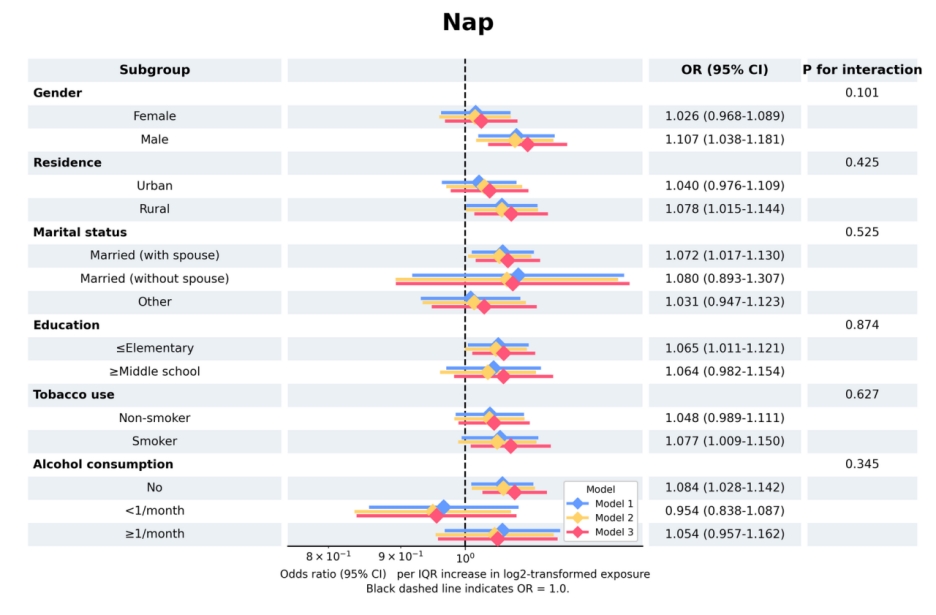


Figure S3. Subgroup analyses of the association between ambient particulate-phase naphthalene (Nap) and prevalent non-neoplastic gastrointestinal diseases.


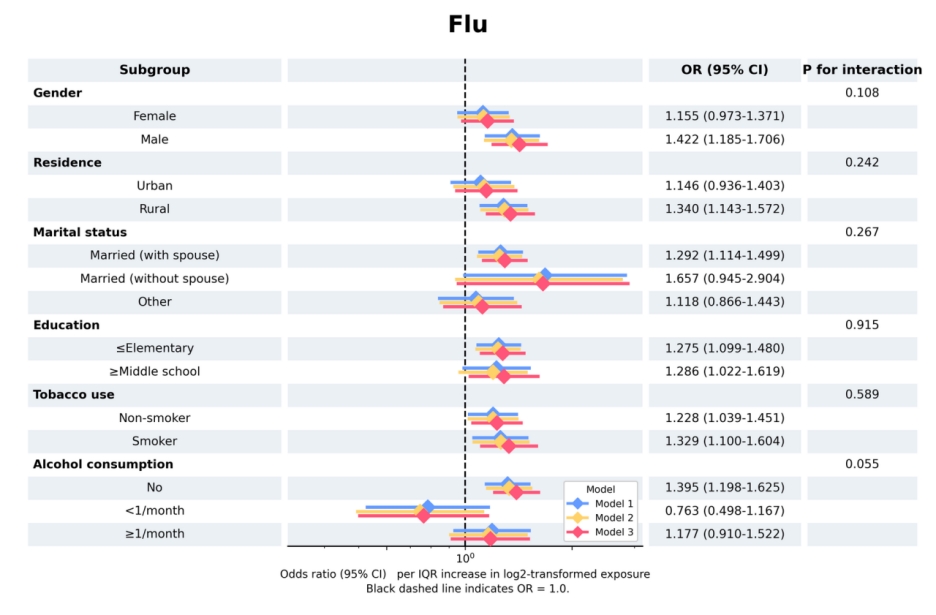


Figure S4. Subgroup analyses of the association between ambient particulate-phase fluorene (Flu) and prevalent non-neoplastic gastrointestinal diseases.


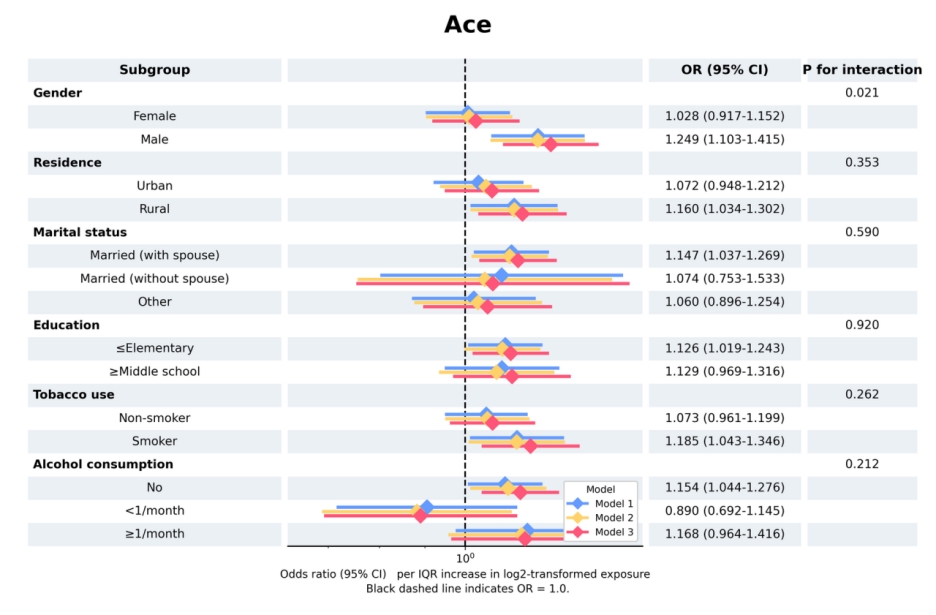


Figure S5. Subgroup analyses of the association between ambient particulate-phase acenaphthene (Ace) and prevalent non-neoplastic gastrointestinal diseases.


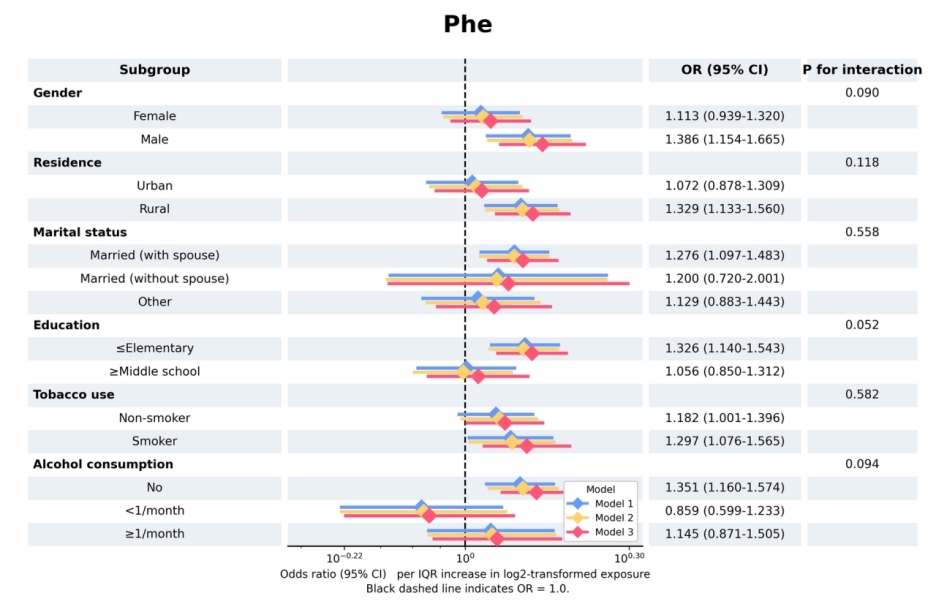


Figure S6. Subgroup analyses of the association between ambient particulate-phase phenanthrene (Phe) and prevalent non-neoplastic gastrointestinal diseases.


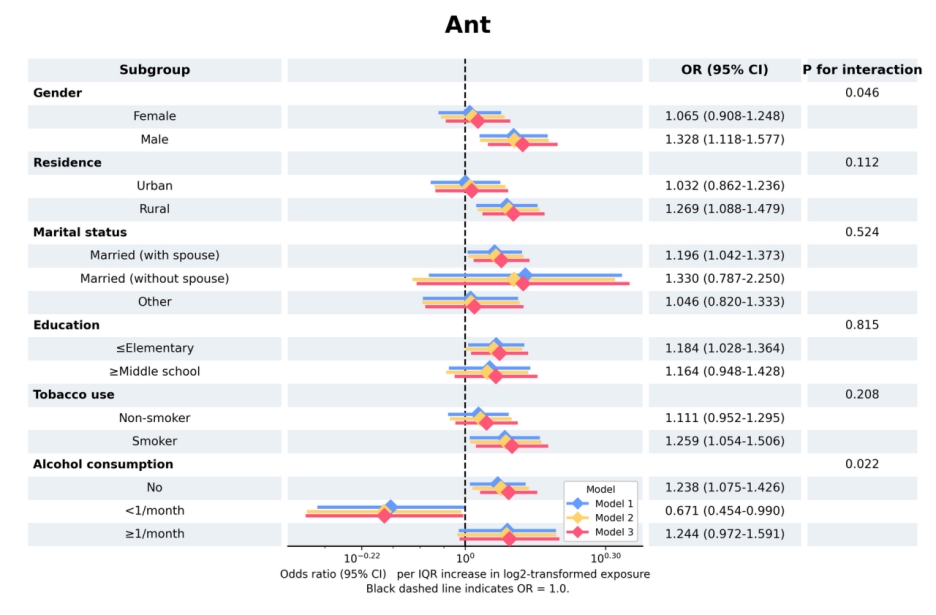


Figure S7. Subgroup analyses of the association between ambient particulate-phase anthracene (Ant) and prevalent non-neoplastic gastrointestinal diseases.


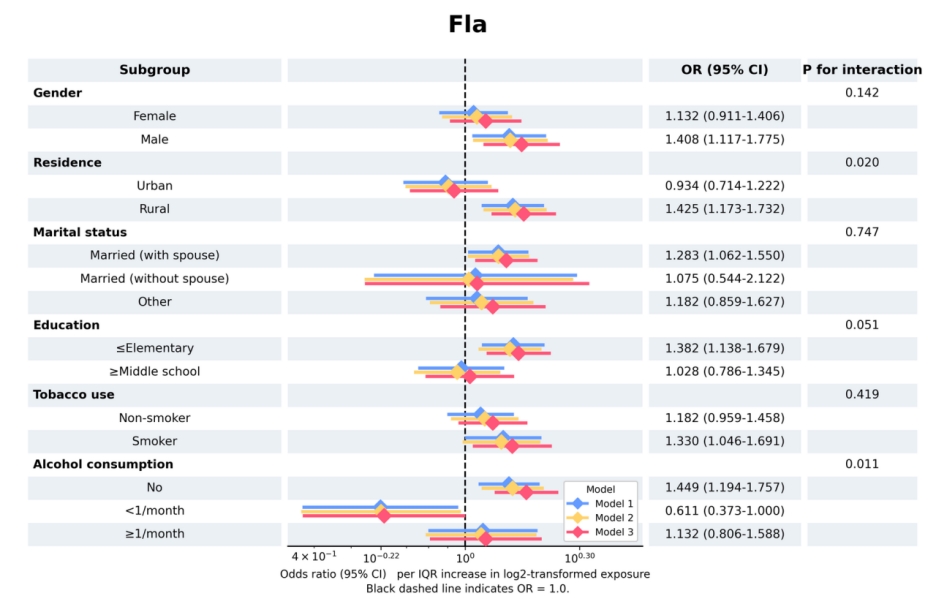


Figure S8. Subgroup analyses of the association between ambient particulate-phase fluoranthene (Fla) and prevalent non-neoplastic gastrointestinal diseases.


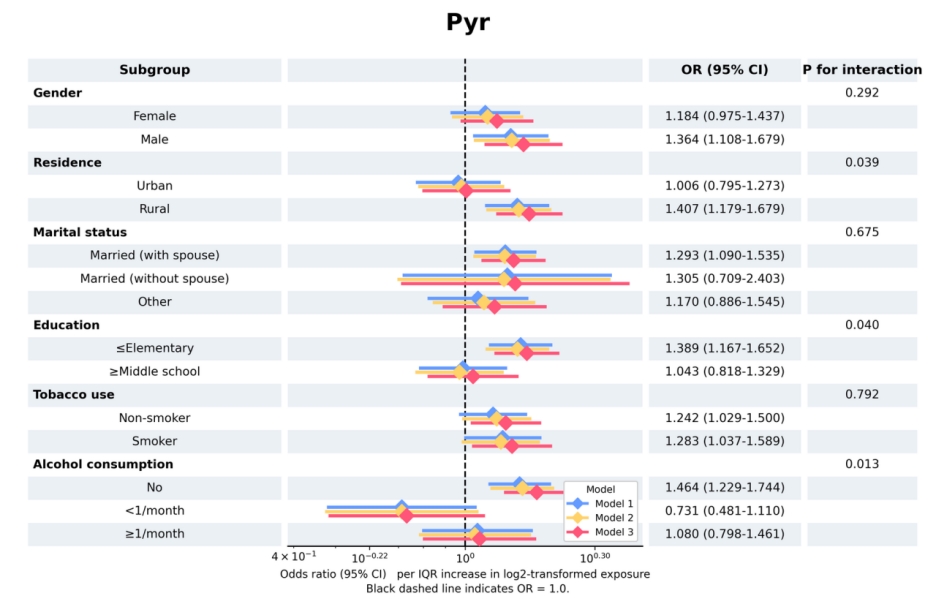


Figure S9. Subgroup analyses of the association between ambient particulate-phase pyrene (Pyr) and prevalent non-neoplastic gastrointestinal diseases.


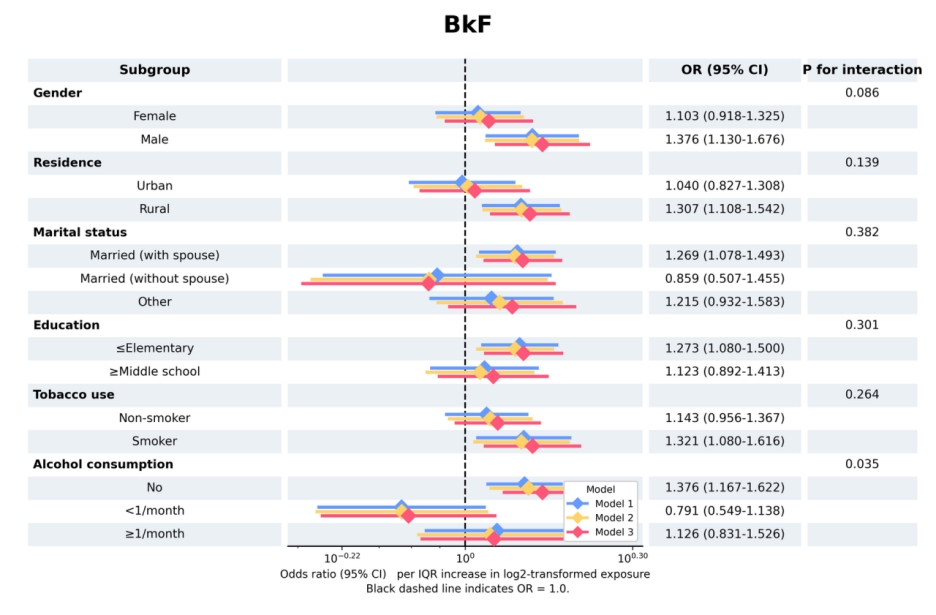


Figure S10. Subgroup analyses of the association between ambient particulate-phase benzo[k]fluoranthene (BkF) and prevalent non-neoplastic gastrointestinal diseases.


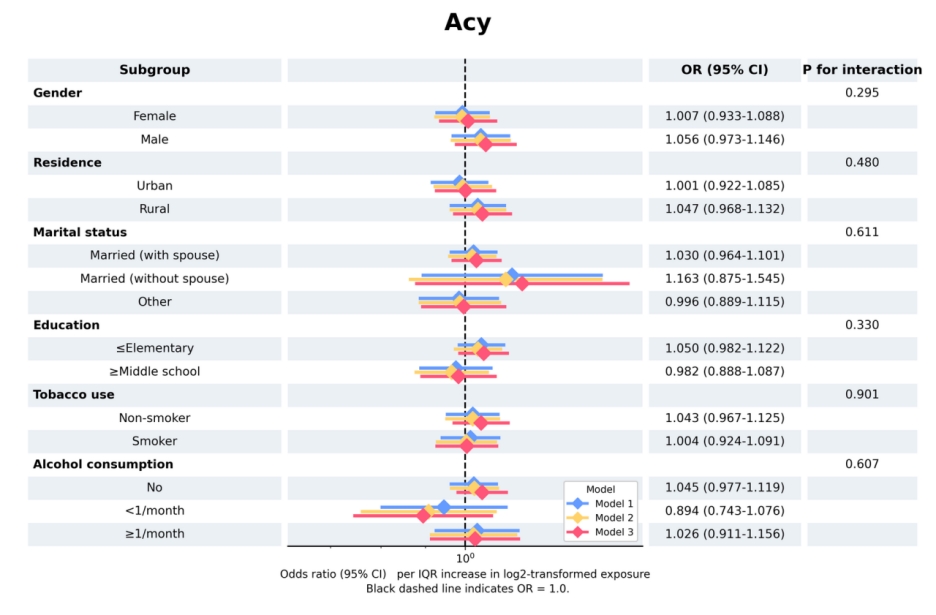


Figure S11. Subgroup analyses of the association between ambient particulate-phase acenaphthylene (Acy) and prevalent non-neoplastic gastrointestinal diseases.


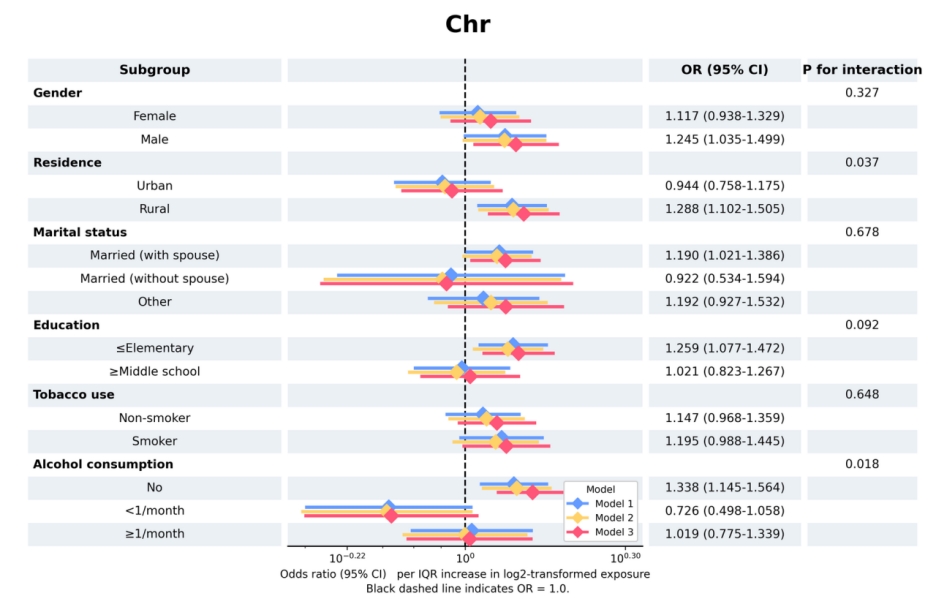


Figure S12. Subgroup analyses of the association between ambient particulate-phase chrysene (Chr) and prevalent non-neoplastic gastrointestinal diseases.


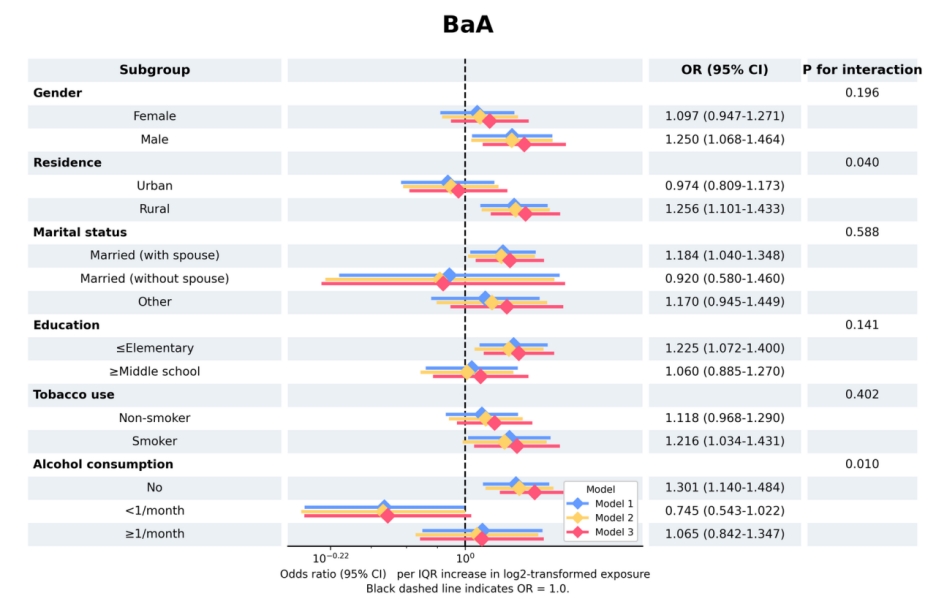


Figure S13. Subgroup analyses of the association between ambient particulate-phase benzo[a]anthracene (BaA) and prevalent non-neoplastic gastrointestinal diseases.


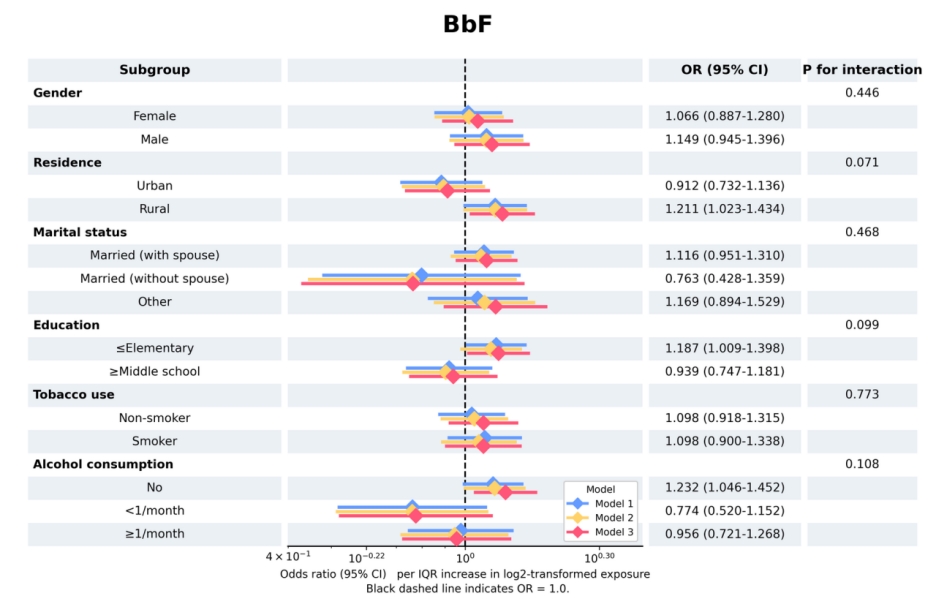


Figure S14. Subgroup analyses of the association between ambient particulate-phase benzo[b]fluoranthene (BbF) and prevalent non-neoplastic gastrointestinal diseases.


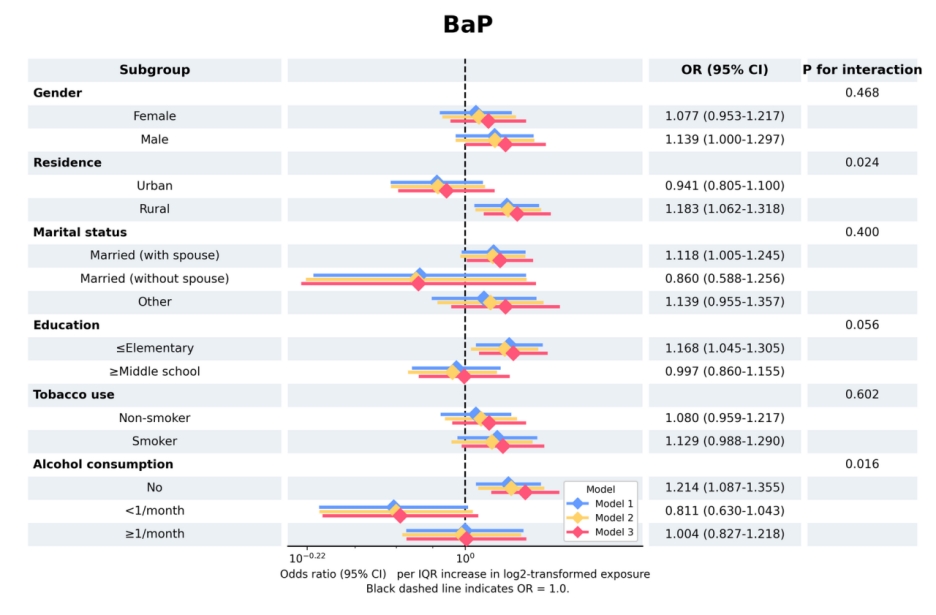


Figure S15. Subgroup analyses of the association between ambient particulate-phase benzo[a]pyrene (BaP) and prevalent non-neoplastic gastrointestinal diseases.


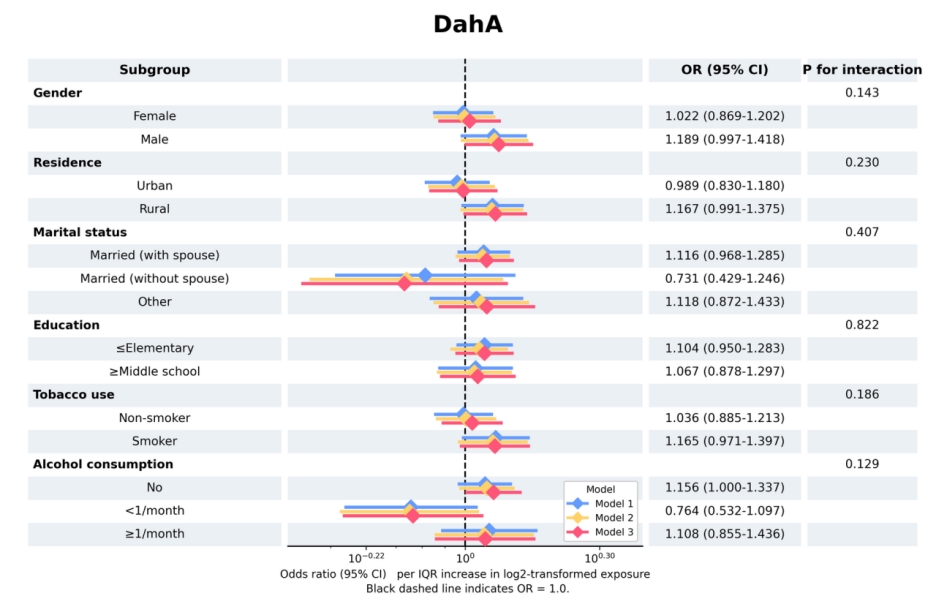


Figure S16. Subgroup analyses of the association between ambient particulate-phase dibenzo[a,h]anthracene (DahA) and prevalent non-neoplastic gastrointestinal diseases.


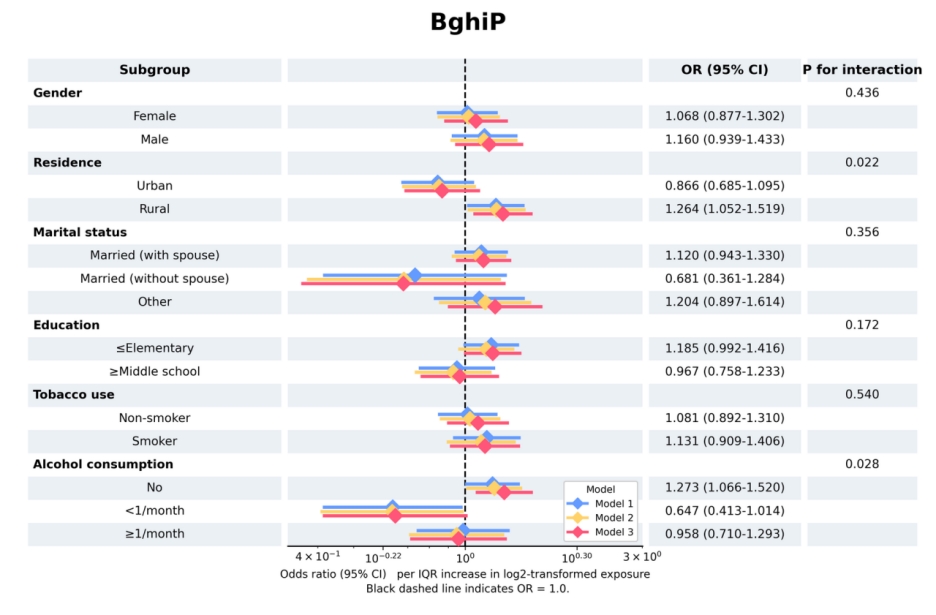


Figure S17. Subgroup analyses of the association between ambient particulate-phase benzo[g,h,i]perylene (BghiP) and prevalent non-neoplastic gastrointestinal diseases.


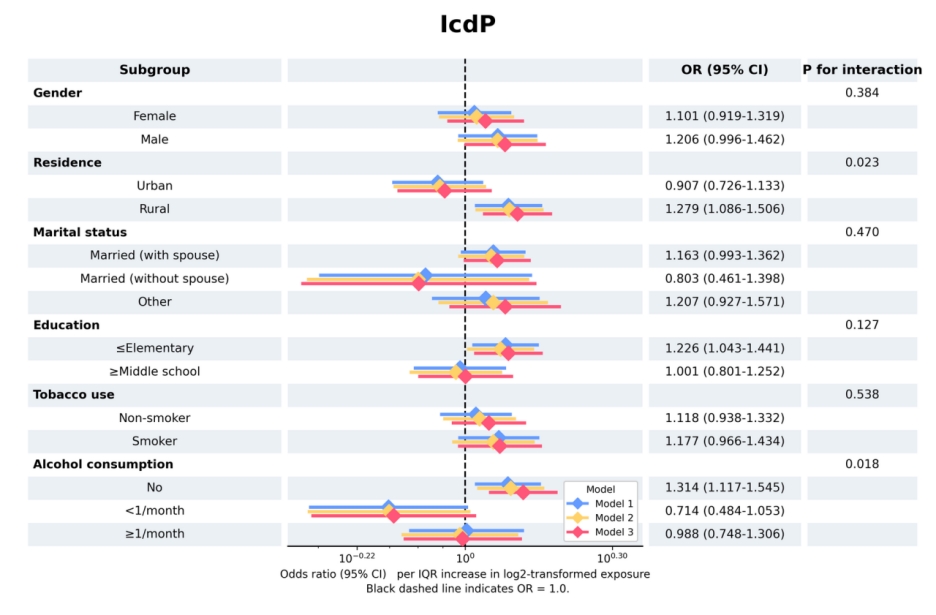


Figure S18. Subgroup analyses of the association between ambient particulate-phase indeno[1,2,3-cd]pyrene (IcdP) and prevalent non-neoplastic gastrointestinal diseases.
